# Partial order among the 14 Bravais types of lattices: basics and applications

**DOI:** 10.1107/S2053273314027351

**Published:** 2015-01-29

**Authors:** Hans Grimmer

**Affiliations:** a Research with Neutrons and Muons, Paul Scherrer Institut, WHGA/342, Villigen PSI, CH-5232, Switzerland

**Keywords:** Bravais lattices, *translationengleiche* subgroups, phase transitions

## Abstract

Neither *International Tables for Crystallography* (ITC) nor available crystallography textbooks state explicitly which of the 14 Bravais types of lattices are special cases of others, although ITC contains the information necessary to derive the result in two ways, considering either the symmetry or metric properties of the lattices. The first approach is presented here for the first time, the second has been given by Michael Klemm in 1982. Metric relations between conventional bases of special and general lattice types are tabulated and applied to continuous equi-translation phase transitions.

## Abstract 2

The partial order among Bravais types of lattices obtained by considering special cases is derived from their space-group symmetry and applied to continuous equi-translation phase transitions.

## 1. Introduction   

**Figure 1:**
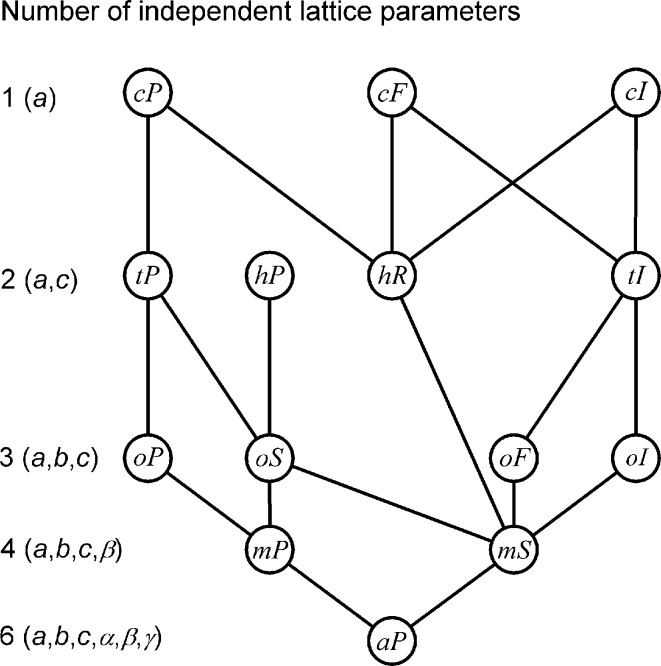
The lattice type of the three-dimensional lattice at the upper end of a line is a special case of the type at its lower end.

The mathematician Michael Klemm (1982 ▶) published a text ‘*Symmetrien von Ornamenten und Kristallen*’ intended for students and lecturers of mathematics. Considering conventional cells for the 14 types of Bravais lattices, he determined what lattice types are special cases of others and illustrated the result in a figure. This result is of basic importance but it is mentioned neither in Volume A of *International Tables for Crystallography* (Hahn, 2002 ▶), which we shall refer to as ITC-A, nor in any of the standard crystallography textbooks. To improve general understanding, Grimmer & Nespolo (2006 ▶) gave a figure where the result was shown using standard crystallographic notations for the lattice types. Klemm (1982 ▶) and Grimmer & Nespolo (2006 ▶) arranged the lattice types on five levels, which correspond to different numbers of independent lattice parameters, as shown in Fig. 1 ▶.

Looking at graphical representations of the various lattice types as given *e.g.* in Fig. 9.1.7.1 of ITC-A one finds that certain relations are obvious, *e.g.* the relations between the primitive lattices *aP* → *mP* → *oP* → *tP* → *cP*, where the arrow points from general to special case. However, there are also pitfalls: (i) *hP* is not a special case of *oP*, although the holohedry of *hP* contains the one of *oP*. (ii) The centred monoclinic lattice type *mS* is called *mC* in Fig. 9.1.7.1. However, *C* centring a lattice of type *mP* gives a new type only if the unique monoclinic axis is not **c**, otherwise it remains of type *mP*.

Unfortunately, misunderstandings of the relations between lattice types seem to be frequent. Grimmer & Nespolo’s (2006 ▶) figure has not had the required effect. This led the author to investigate other ways of deriving the result. Considering Niggli-reduced primitive cells instead of the conventional centred ones did not appear advantageous. Finally, the author found that ITC-A contains results that allow one to derive the relations in two different ways, one based on the metric properties of lattices, the other on their symmetries.

In §2 it will be shown how Fig. 1 ▶ can be obtained from metric results given in ITC-A. §3 presents for the first time the approach based on the space-group symmetry of the lattice types. In retrospect, it may be surprising that this approach has not been published earlier, considering that it is based on results available already in *Internationale Tabellen zur Bestimmung von Kristallstrukturen* (Hermann, 1935 ▶). These tables list the *translationengleiche* (*i.e.* equi-translation) subgroups of the space groups in three dimensions, based on the work of Hermann (1929 ▶). In §4 metric relations between conventional bases of special and minimally more general lattice types are tabulated. They are applied to continuous equi-translation phase transitions in §5.

## 2. Metric-based derivation of the partial order among the 14 lattice types   

Similar to Klemm (1982 ▶), Gruber (2002 ▶) gives in Table 9.3.4.1 of ITC-A necessary and sufficient conditions for conventional cells of lattices belonging to one of the 14 lattice types. Except for *hP* and the three cubic types, the conditions contain at least one ‘<’ or ‘≠’. Replacing one of them in turn by ‘=’, the Bravais types that are minimal special cases of the given type can be determined. In the more complicated cases, Gruber (2002 ▶) does this already in footnotes to his table. Let us do it in all cases.

There are no special cases of *cP*, *cI*, *cF* (*a* = *b* = *c*, α = β = γ = 90°) and of *hP* (*a* = *b*, α = β = 90°, γ = 120°).

For the primitive conventional tetragonal cell the conditions for *tP* are according to Table 9.3.4.1: *a* = *b* ≠ *c*, α = β = γ = 90°. If *b* = *c* the type is *cP*.

For the body-centred conventional tetragonal cell the conditions for *tI* are: 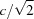 ≠ *a* = *b* ≠ *c*, α = β = γ = 90°. If 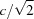 = *a* the type is *cF*, if *b* = *c* the type is *cI*.

For the primitive conventional orthorhombic cell the conditions for *oP* are: *a* < *b* < *c*, α = β = γ = 90°. If *a* = *b* or *b* = *c* the type is *tP*.

For the body-centred conventional orthorhombic cell the conditions for *oI* are: *a* < *b* < *c*, α = β = γ = 90°. If *a* = *b* or *b* = *c* the type is *tI*.

For the all-face-centred conventional orthorhombic cell the conditions for *oF* are: *a* < *b* < *c*, α = β = γ = 90°. If *a* = *b* or *b* = *c* the type is *tI*.

For the *C*-face-centred conventional orthorhombic cell the conditions for *oC* are: *a* < *b* ≠ 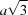, α = β = γ = 90°. If *a* = *b* the type is *tP*, if *b* = 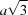 the type is *hP*.

For the primitive conventional rhombohedral cell the conditions for *hR* are: *a* = *b* = *c*, α = β = γ, α ≠ 60°, α ≠ 90°, α ≠ arccos(−1/3) = 109°28′16″. If α = 60° the type is *cF*, if α = 90° the type is *cP*, if α = arccos(−1/3) the type is *cI*.

For the primitive conventional monoclinic cell (unique axis *b*) the conditions for *mP* are: −2*c* cosβ < *a* < *c*, α = γ = 90° < β. If β = 90° the type is *oP*, if −2*c* cosβ = *a* or *a* = *c* the type is *oS*.

For the body-centred conventional monoclinic cell (unique axis *b*) the conditions for *mI* are: −*c* cosβ < *a* < *c*, α = γ = 90° < β and additional conditions given by Gruber that exclude *hR*. If β = 90° the type is *oI*, if −*c* cosβ = *a* the type is *oC*, if *a* = *c* the type is *oF*.

These considerations show that all types with less than four independent lattice parameters are special cases of at least one of the two monoclinic types. Fig. 1 ▶ follows because, obviously, the two monoclinic types are special cases of the anorthic (= triclinic) one.

## 3. Symmetry-based derivation of a partial order among the 14 lattice types   

Each lattice type can be characterized by the space-group type to which its lattices belong. These types are symmorphic and possess the point group of the holohedry. Table 1 ▶ gives the corresponding information, which can also be found in Vainshtein (1981 ▶) and in Borchardt-Ott (1997 ▶).

The notations *mS* and *oS*, where *S* stands for ‘side-face-centred’ (*i.e. seitenflächenzentriert*), have been proposed as standard ones by de Wolff *et al.* (1985 ▶). Note that Gruber (2002 ▶) uses a body-centred (*innenzentriert*) cell *mI*.

To answer the question ‘*Which lattice types are special cases of others*?’ we shall make use of information given in ITC-A or, in more detail, in Volume A1 of *International Tables for Crystallography* (Wondratschek & Müller, 2004 ▶).

Consider a lattice type *g* and let *G* be the corresponding space-group type. Find for *G* those maximal *translation­engleiche* (*i.e.* type I) subgroup types that occur in Table 1 ▶. Examples:

(i) *g* = *oC*. *P*2/*m* appears once and *C*2/*m* twice in the list of maximal subgroups of type I of *G* = *Cmmm*.

(ii) *g* = *cP*. A set of three conjugate *P*4/*mmm* and a set of four conjugate *R* 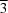 *m* appear in the list of maximal subgroups of type I of *G* = *Pm* 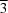 *m*.

**Figure 2:**
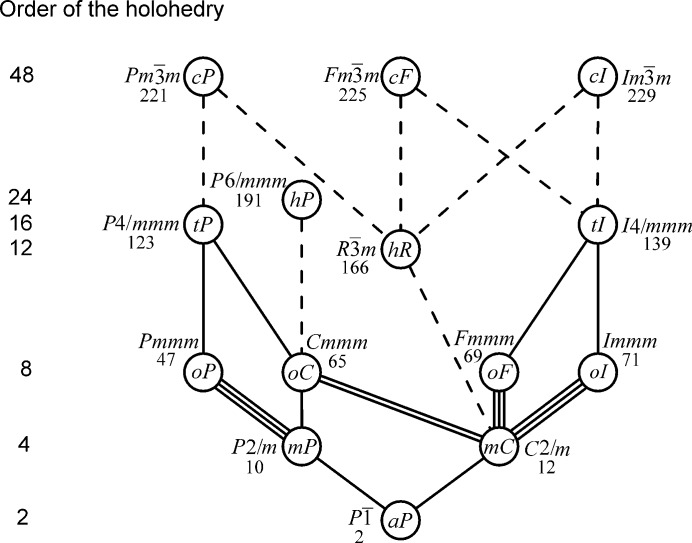
The Bravais type of the three-dimensional lattice at the upper end of a line is a special case of the type at its lower end. Solid lines indicate normal subgroups, dashed lines sets of conjugate subgroups. The number of conjugate groups in a set is equal to the subgroup index, *i.e.* the quotient of the orders of the corresponding point groups (4 for the transition cubic to rhombohedral and 3 in the other cases).

This procedure leads to the result shown in Fig. 2 ▶.

For discussing the numbers of subgroups shown in Fig. 2 ▶, the definition of conventional cells, as given in Part 2 of ITC-A, is needed. This is shown in Table 2 ▶ for lattices in three dimensions.

Alternatively, two lattice systems, hexagonal and rhombohedral, are combined in the hexagonal crystal family, where *hR* is considered as a rhombohedrally centred hexagonal lattice instead of a primitive rhombohedral lattice.

Fig. 2 ▶ shows the changes of lattice types that are possible in phase transitions where the lattice changes continuously: the corresponding pairs of lattice types are connected by lines. Note that the two types of these pairs always belong to different crystal families, whence they have different conventional bases.

Let us now discuss the numbers of subgroups shown in Fig. 2 ▶.

A rhombohedral deformation of the conventional cubic cell lets only one of the four threefold axes survive, transforming the lattice types *cP*, *cF* and *cI* into *hR*. A tetragonal deformation of the conventional cubic cell lets only one of the three fourfold axes survive, transforming *cP* into *tP*, *cF* and *cI* into *tI*.

The plane perpendicular to the sixfold axis of a lattice of type *hP* contains three pairs of mutually orthogonal twofold axes. An orthorhombic deformation lets only one of these three pairs survive, transforming *hP* into *oC*.

A plane perpendicular to the fourfold axis of a tetragonal lattice cuts the conventional tetragonal cell into a square, which contains two pairs of mutually orthogonal twofold axes, parallel either to the edges or the diagonals of the square. An orthorhombic deformation lets only one of these two pairs survive, transforming *tP* either into *oP* or *oC*, and *tI* either into *oI* or *oF*, depending on whether the square is deformed into a rectangle or a rhombus.

Perpendicular to the threefold axis in a primitive rhombohedral cell, there are three twofold axes at 120° one to another. A monoclinic deformation of this cell can be done in three ways, preserving one of the twofold axes and transforming *hR* into *mC*.

A monoclinic deformation of the conventional ortho­rhombic cell preserves only one of the three mutually perpendicular twofold orthorhombic axes. In all three cases *oP* is transformed into *mP* whereas *oF* and *oI* are transformed into *mC*. The type *oC* is transformed into *mP* if the twofold axis perpendicular to the *C*-face survives, into *mC* if one of the two twofold axes in the *C*-face survives.

Finally, an anorthic deformation removes the twofold monoclinic axis and transforms *mP* and *mC* into *aP*.

**Figure 3:**
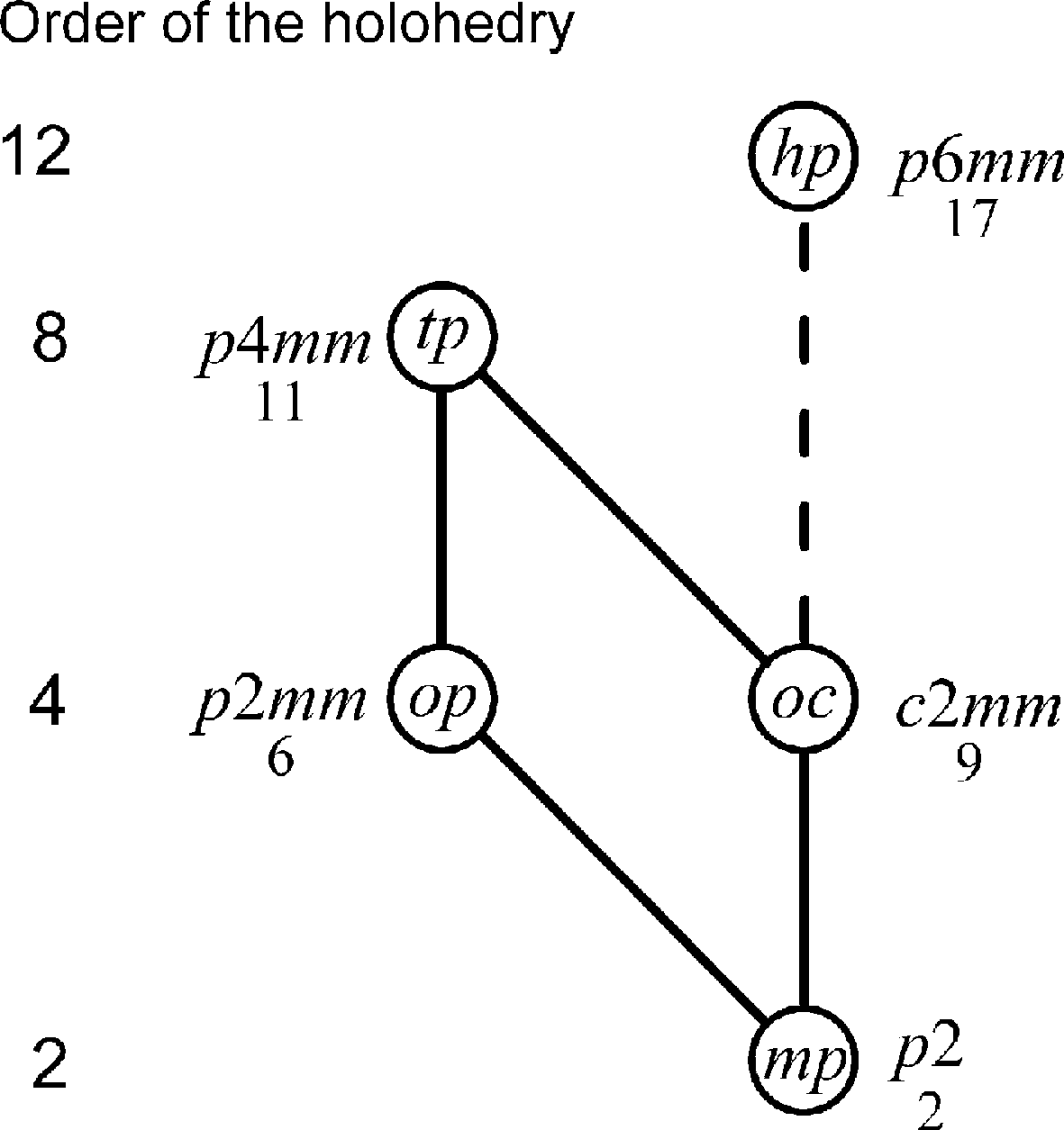
The type of the two-dimensional lattice at the upper end of a line is a special case of the type at its lower end. Solid lines indicate normal subgroups, dashed lines sets of conjugate subgroups.

In two dimensions one obtains Fig. 3 ▶.

Note that the partially ordered set formed by the two-dimensional lattice types *mp*, *op*, *oc*, *tp* and *hp* has the same structure as the partially ordered set formed by *mP*, *oP*, *oC*, *tP* and *hP*, as indicated by the notation.

## 4. Metric relations between conventional bases of ‘neighbouring’ lattice types   

In this section we express the conventional basis of each lattice type in terms of the conventional basis of each lattice type that is minimally more general. In the case of rhombohedral lattices both conventions are considered in Table 3 ▶, the one where a primitive cell is used for *hR* and the one where a rhombohedrally centred hexagonal cell is used in the usual obverse setting with lattice points at 0, 0, 0, 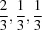 and 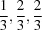.

In the case of conjugate subgroups the various expressions for **a**′, **b**′, **c**′ are equivalent by symmetry. For the transitions *hR* to cubic, the three other possibilities are obtained from the first one keeping one of the vectors **a**′, **b**′, **c**′ and changing the signs of the other two. For the transitions tetragonal to cubic, the three possibilities are related by cyclic permutations **a**′ → **b**′ → **c**′ → **a**′; for the transition *oS* to *hP* they are related by cyclic permutations **a**′ → **b**′ → **d**′ → **a**′. Note that for the transition *mS* to *hR* the three possibilities are related by cyclic permutations **a**′ → **b**′ → **c**′ → **a**′ if *hR* is considered as a primitive rhombohedral lattice, and by **a**′ → **b**′ → **d**′ → **a**′ if *hR* is considered as a rhombohedrally centred hexagonal lattice.

The transition *mS → hR* is given (for *hR*, hex and *hR*, rho) first with unique monoclinic axis **b**, then with axis **c**.

The column ‘Det’ in Tables 3 ▶ and 4 ▶ gives the determinant of the matrix **M** expressing **a**′, **b**′, **c**′ in terms of **a**, **b**, **c**. It equals the number of lattice points in a conventional cell of L_2_ divided by the number of lattice points in a conventional cell of L_1_.

The restrictions given in Table 2 ▶ do not determine a unique conventional basis. In accordance with Volume A1 of *International Tables for Crystallography* (Wondratschek & Müller, 2004 ▶), conventional bases for lattice-type pairs L_1_ and L_2_ have been chosen in Tables 3 ▶ and 4 ▶ such that the matrix **M** becomes as simple as possible (*e.g.* **M** is the identity matrix for each of the transitions *aP* → *mP* → *oP* → *tP* → *cP*). As a consequence, the conditions given in the column ‘Limiting case of L_1_ for which it becomes L_2_’ of Tables 3 ▶ and 4 ▶ take a particularly simple form. This column contains two conditions that must be satisfied for the transitions *mS* → *hR* and anorthic → monoclinic, in accordance with Fig. 1 ▶, which shows that the number of independent lattice parameters is reduced by 2 in these cases.

Remark to Table 4 ▶: for the transitions monoclinic to orthorhombic, the unique monoclinic axis is the orthorhombic axis **c**′ in the first, **b**′ in the second and **a**′ in the third line; for the transitions anorthic to monoclinic, the unique monoclinic axis is **b**′ in the first line, **c**′ in the second.

## 5. Applications   

Let us consider two applications to continuous equi-translation phase transitions. Both concern transitions *mS* ↔ *hR*, *hR* being considered as a rhombohedrally centred hexagonal lattice in the first example and as a primitive rhombohedral lattice in the second.

(i) Przeniosło *et al.* (2014 ▶) measured the monoclinic deformation of the crystal lattice of hematite (α-Fe_2_O_3_) at room temperature. Hematite is paramagnetic with space group *R* 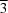 *c* above its Néel temperature *T*_N_ = 955 K. Below *T*_N_ it is weakly ferromagnetic (canted antiferromagnet) with space group *C*2/*c* down to the Morin temperature *T*_M_ = 260 K. The lattice type is therefore *mC* at room temperature and changes to *hR* at *T*_N_. Equations A1–A3 in Appendix *A* of Przeniosło *et al.* (2014 ▶) show that they chose a conventional *C*-centred monoclinic cell with basis **a** = 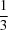
(−**a**′ + **b**′ − 2**c**′), **b** = −**a**′ − **b**′, **c** = **c**′, where **a**′, **b**′, **c**′ is the conventional basis of the rhombo­hedrally centred hexagonal cell, as suggested in our Table 3 ▶. The authors found for their sample I at room temperature: *a* = 961.935 (12), *b* = 503.575 (7), *c* = 1375.277 (17) pm and β = 162.4049 (2)°. Neglecting experimental uncertainties, we obtain from the last column of Table 3 ▶ that the lattice becomes rhombohedral if β = arccos[−2*c*/(3*a*)] = 162.3889° and *a* = 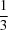
(3*b* ^2^ + 4*c* ^2^)^1/2^ = 961.845 pm.

It follows that although the measurement was performed approximately 660 K below the phase-transition temperature, *a* deviates by only 0.1 pm and β by only 1 minute of arc from the values for a rhombohedral lattice. The calculations given in Table 2 ▶ of Przeniosło *et al.* (2014 ▶), which take account of experimental uncertainties, lead to a similar deviation for *a* and an even smaller deviation for β of the order of 1 second of arc. For their sample VI, the deviation for β even has opposite sign. We conclude that the magnetic ordering, which destroys the trigonal symmetry, affects the lattice parameters so little that high-resolution synchrotron radiation diffraction is necessary to measure the effect.

(ii) Pyridinium tetrafluoroborate [C_5_H_6_N]^+^BF_4_^−^ has been investigated by Czarnecki *et al.* (1998 ▶). It is paraelectric at room temperature with space group *R* 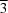 *m* and undergoes at *T* = 238.7 K a continuous transition to a ferroelectric phase with space group *C*2. It follows that the lattice type is *hR* at room temperature and changes to *mC* at *T*. Using high-resolution neutron powder diffraction, Czarnecki *et al.* (1998 ▶) found for the conventional primitive rhombohedral cell **a**′, **b**′, **c**′ at 293 K: |**a**′| = |**b**′| = |**c**′| = *a*′ = 567.074 (7) pm and α′ = β′ = γ′ = 97.305 (1)°. The entry at the bottom of our Table 3 ▶ tells us that *C*-centring the conventional monoclinic cell with basis **a** = −**a**′ − **c**′, **b** = −**a**′ + **c**′, **c** = **a**′ + **b**′ + **c**′ produces a primitive cell that coincides with the rhombohedral one in the limiting case that *mC* becomes *hR*. According to their Fig. 3 ▶, Czarnecki *et al.* (1998 ▶) chose a different conventional monoclinic cell 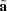 = **a**′ + **c**′, 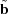 = **a**′ − **c**′, 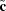 = **b**′. They found at 230 K: 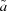 = 734.68 (2), 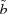 = 839.95 (2), 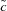 = 571.14 (2) pm and 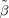 = 101.952 (2)°. With our cell choice **a** = −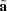, **b** = −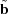, **c** = 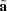 + 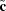, we obtain at 230 K: *a* = 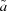, *b* = 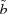, *c* = 831.96 pm and β = 137.808°. It follows that arccos[−2*c*/(3*a*)] − β = 1.212° and 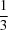
[3*b* ^2^ + 4*c* ^2^]^1/2^ − *a* = 2.07 pm, showing that 8.7 K below the transition temperature *T* the lattice already differs considerably from a rhombohedral one, in contrast to the first example.

## 6. Discussion and conclusions   

Figs. 1 ▶ ▶–3 ▶ show basic relations between the lattice types. These relations are of importance also for applications: they tell us the changes of lattice type possible in continuous equi-translation phase transitions, as discussed *e.g.* by Landau & Lifshitz (1980 ▶) or Burns & Glazer (1990 ▶). They can be useful also in cases of twinning by ‘metric merohedry’ as defined by Nespolo & Ferraris (2000 ▶). The use of Fig. 2 ▶ and of Tables 3 ▶ and 4 ▶, which give metric relations between the conventional bases of lattice types joined by lines in Fig. 2 ▶, has been illustrated with two examples. Tables 3 ▶ and 4 ▶ also illustrate how the distinction between conjugate and normal subgroups made in Fig. 2 ▶ affects the relation between the conventional bases.

For all these reasons, it is suggested that figures like Figs. 2 ▶ and 3 ▶ and tables like Tables 3 ▶ and 4 ▶ be introduced in future editions of ITC-A. Symmetry aspects are central to ITC-A; its Fig. 10.1.3.2 and Fig. 10.1.3.1 show relations between the types of crystallographic point groups in three and two dimensions in a similar way as our Figs. 2 ▶ and 3 ▶ give relations between the lattice types. To balance the information given in ITC-A on the point group and lattice aspects of the space groups, also the Bravais lattice type should be given in the banner line of each space-group type.

## Figures and Tables

**Table 1:** The 14 lattice types and the corresponding space-group types (SGTs)

Lattice type	International SGT symbol	Schoenflies SGT symbol	Number of SGT	Order of the holohedry
*aP*	*P* 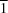	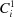	2	2
*mP*	*P*2/*m*	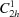	10	4
*mS* (*mA*, *mB*, *mC*, *mI*)	*C*2/*m*	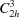	12	4
*oP*	*Pmmm*	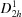	47	8
*oS* (*oA*, *oB*, *oC*)	*Cmmm*	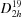	65	8
*oF*	*Fmmm*	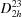	69	8
*oI*	*Immm*	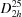	71	8
*tP*	*P*4/*mmm*	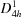	123	16
*tI*	*I*4/*mmm*	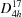	139	16
*hR*	*R* 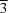 *m*	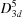	166	12
*hP*	*P*6/*mmm*	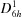	191	24
*cP*	*Pm* 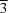 *m*	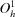	221	48
*cF*	*Fm* 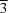 *m*	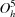	225	48
*cI*	*Im* 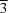 *m*	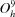	229	48

**Table 2:** Conventional bases for the seven lattice systems Note that Gruber (2002 ▶) used stricter conventions, which generally depend also on the lattice type, *e.g. a* *b* *c* for *oP*, *oF* and *oI*.

Crystal family	Lattice system	Holohedry	Restrictions	Free parameters	Lattice types
Cubic	Cubic	*m* 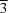 *m*	|**a**| = |**b**| = |**c**| = *a*, = = = 90	*a*	*cP*, *cF*, *cI*
Tetragonal	Tetragonal	4/*mmm*	|**a**| = |**b**| = *a*, = = = 90	*a*, *c*	*tP*, *tI*
Hexagonal	Hexagonal	6/*mmm*	|**a**| = |**b**| = *a*, = = 90, = 120	*a*, *c*	*hP*
	Rhombohedral	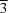 *m*	|**a**| = |**b**| = |**c**| = *a*, = =	*a*,	*hR*
Orthorhombic	Orthorhombic	*mmm*	= = = 90	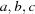	*oP*, *oS*, *oF*, *oI*
Monoclinic	Monoclinic	2/*m*	= = 90 (**b** unique)	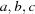 ,	*mP*, *mS*
			= = 90 (**c** unique)	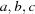 ,	
Anorthic	Anorthic	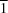		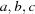 , , ,	*aP*

**Table 3:** Metric relations between the conventional unit cells of lattice types related by conjugate subgroups, *i.e.* joined by dashed lines in Fig. 2 ▶ ‘*hR*, hex’ considers *hR* as a rhombohedrally centred hexagonal lattice, ‘*hR*, rho’ considers *hR* as a primitive rhombohedral lattice; **d** = **a** **b**.

Lattice types L_1_ L_2_	Corresponding space-group types	Basis **a**, **b**, **c** of conventional cell for L_2_ expressed in terms of basis **a**, **b**, **c** of conventional cell for L_1_				Det	Limiting case of L_1_ for which it becomes L_2_	Basis **a**, **b**, **c** of conventional cell for L_1_ expressed in terms of basis **a**, **b**, **c** of conventional cell for L_2_		
		**a**	**b**	**d**	**c**			**a**	**b**	**c**
*hR*, hex *cP*	*R* 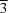 *m* *Pm* 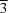 *m*	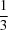 (2**a**+**b**+**c**)	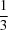 (**a**+**b**+**c**)		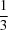 (**a**+2**b** **c**)	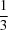	*c* = (6^1/2^) *a*	**a** **b**	**b** **c**	**a**+**b**+**c**
		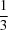 (2**a**+**b**+**c**)	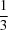 (**a**+**b**+**c**)		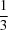 (**a**+2**b** **c**)			**a**+**b**	**b**+**c**	**a** **b** **c**
		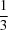 (2**a**+**b**+**c**)	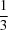 (**a**+**b**+**c**)		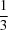 (**a**+2**b** **c**)			**a** **b**	**b**+**c**	**a**+**b** **c**
		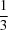 (2**a**+**b**+**c**)	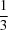 (**a**+**b**+**c**)		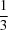 (**a**+2**b** **c**)			**a**+**b**	**b** **c**	**a** **b**+**c**
*hR*, hex *cF*	*R* 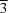 *m* *Fm* 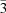 *m*	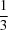 (4**a**+2**b** **c**)	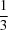 (2**a**2**b**+**c**)		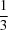 (2**a**+4**b**+**c**)	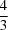	*c* = 6^1/2^ *a*	(**a**+**b**)	(**b**+**c**)	**a**+**b**+**c**
		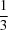 (4**a**+2**b** **c**)	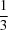 (2**a**2**b**+**c**)		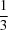 (2**a**+4**b**+**c**)			(**a** **b**)	(**b** **c**)	**a** **b** **c**
		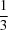 (4**a**+2**b** **c**)	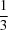 (2**a**2**b**+**c**)		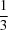 (2**a**+4**b**+**c**)			(**a**+**b**)	(**b** **c**)	**a**+**b** **c**
		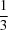 (4**a**+2**b** **c**)	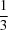 (2**a**2**b**+**c**)		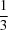 (2**a**+4**b**+**c**)			(**a** **b**)	(**b**+**c**)	**a** **b**+**c**
*hR*, hex *cI*	*R* 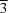 *m* *Im* 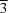 *m*	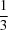 (2**a**+**b**2**c**)	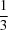 (**a** **b**+2**c**)		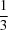 (**a**+2**b**+2**c**)	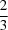	*c* = (6^1/2^) *a*	**a**+**b**	**b**+**c**	(**a**+**b**+**c**)
		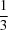 (2**a**+**b**2**c**)	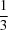 (**a** **b**+2**c**)		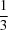 (**a**+2**b**+2**c**)			**a** **b**	**b** **c**	(**a** **b** **c**)
		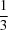 (2**a**+**b**2**c**)	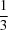 (**a** **b**+2**c**)		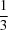 (**a**+2**b**+2**c**)			**a**+**b**	**b** **c**	(**a**+**b** **c**)
		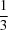 (2**a**+**b**2**c**)	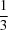 (**a** **b**+2**c**)		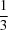 (**a**+2**b**+2**c**)			**a** **b**	**b**+**c**	(**a** **b**+**c**)
*hR*, rho *cP*	*R* 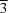 *m* *Pm* 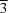 *m*	**a**	**b**		**c**	1	cos = 0 = 90	**a**	**b**	**c**
		**a**	**b**		**c**			**a**	**b**	**c**
		**a**	**b**		**c**			**a**	**b**	**c**
		**a**	**b**		**c**			**a**	**b**	**c**
*hR*, rho *cF*	*R* 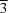 *m* *Fm* 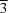 *m*	**a**+**b**+**c**	**a** **b**+**c**		**a**+**b** **c**	4	cos = = 60	(**b**+**c**)	(**c**+**a**)	(**a**+**b**)
		**a**+**b**+**c**	**a**+**b** **c**		**a** **b**+**c**			(**b** **c**)	(**c**+**a**)	(**a** **b**)
		**a** **b** **c**	**a** **b**+**c**		**a** **b**+**c**			(**b** **c**)	(**c** **a**)	(**a**+**b**)
		**a** **b** **c**	**a**+**b** **c**		**a**+**b** **c**			(**b**+**c**)	(**c** **a**)	(**a** **b**)
*hR*, rho *cI*	*R* 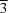 *m* *Im* 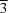 *m*	**b**+**c**	**a**+**c**		**a**+**b**	2	cos = 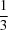 = 109.47	(**a**+**b**+**c**)	(**a** **b**+**c**)	(**a**+**b** **c**)
		**b**+**c**	**a** **c**		**a** **b**			(**a** **b** **c**)	(**a**+**b** **c**)	(**a** **b**+**c**)
		**b** **c**	**a**+**c**		**a** **b**			(**a**+**b** **c**)	(**a** **b** **c**)	(**a**+**b**+**c**)
		**b** **c**	**a** **c**		**a**+**b**			(**a** **b**+**c**)	(**a**+**b**+**c**)	(**a** **b** **c**)
*tP* *cP*	*P*4/*mmm* *Pm* 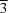 *m*	**a**	**b**		**c**	1	*c* = *a*	**a**	**b**	**c**
		**c**	**a**		**b**			**b**	**c**	**a**
		**b**	**c**		**a**			**c**	**a**	**b**
*tI* *cF*	*I*4/*mmm* *Fm* 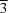 *m*	**a**+**b**	**a**+**b**		**c**	2	*c* = 2^1/2^ *a*	(**a** **b**)	(**a**+**b**)	**c**
		**c**	**a**+**b**		**a**+**b**			(**b** **c**)	(**b**+**c**)	**a**
		**a**+**b**	**c**		**a**+**b**			(**c** **a**)	(**c**+**a**)	**b**
*tI* *cI*	*I*4/*mmm* *Im* 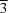 *m*	**a**	**b**		**c**	1	*c* = *a*	**a**	**b**	**c**
		**c**	**a**		**b**			**b**	**c**	**a**
		**b**	**c**		**a**			**c**	**a**	**b**
*oS* *hP*	*Cmmm* *P*6/*mmm*	(**a**+**b**)	(**a** **b**)	**b**	**c**		*a* = 3^1/2^ *b*	**a**+**b**	**d**	**c**
		**b**	(**a**+**b**)	(**a** **b**)	**c**			**b**+**d**	**a**	**c**
		(**a** **b**)	**b**	(**a**+**b**)	**c**			**d**+**a**	**b**	**c**
*mS* *hR*, hex	*C*12/*m*1 *R* 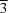 *m*	(3**a**+**b**+2**c**)	(3**a** **b**+2**c**)	**b**	**c**	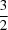	cos = 2*c*/(3*a*) and 9*a* ^2^ = 3*b* ^2^ + 4*c* ^2^	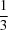 (**a**+**b**2**c**)	**d**	**c**
		**b**	(3**a**+**b**+2**c**)	(3**a** **b**+2**c**)	**c**			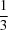 (**b**+**d**2**c**)	**a**	**c**
		(3**a** **b**+2**c**)	**b**	(3**a**+**b**+2**c**)	**c**			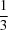 (**d**+**a**2**c**)	**b**	**c**
	*A*112/*m* *R* 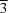 *m*	(3**b**+**c**+2**a**)	(3**b** **c**+2**a**)	**c**	**a**		cos = 2*a*/(3*b*) and 9*b* ^2^ = 3*c* ^2^ + 4*a* ^2^	**c**	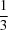 (**a**+**b**2**c**)	**d**
		**c**	(3**b**+**c**+2**a**)	(3**b** **c**+2**a**)	**a**			**c**	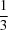 (**b**+**d**2**c**)	**a**
		(3**b** **c**+2**a**)	**c**	(3**b**+**c**+2**a**)	**a**			**c**	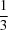 (**d**+**a**2**c**)	**b**
*mS* *hR*, rho	*C*12/*m*1 *R* 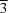 *m*	(**a**+**b**)	**a**+**c**		(**a** **b**)		cos = 2*c*/(3*a*) and 9*a* ^2^ = 3*b* ^2^ + 4*c* ^2^	**a** **c**	**a**+**c**	**a**+**b**+**c**
		(**a** **b**)	(**a**+**b**)		**a**+**c**			**b** **a**	**b**+**a**	**a**+**b**+**c**
		**a**+**c**	(**a** **b**)		(**a**+**b**)			**c** **b**	**c**+**b**	**a**+**b**+**c**
	*A*112/*m* *R* 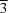 *m*	(**b**+**c**)	**a**+**b**		(**b** **c**)		cos = 2*a*/(3*b*) and 9*b* ^2^ = 3*c* ^2^ + 4*a* ^2^	**a**+**b**+**c**	**a** **c**	**a**+**c**
		(**b** **c**)	(**b**+**c**)		**a**+**b**			**a**+**b**+**c**	**b** **a**	**b**+**a**
		**a**+**b**	(**b** **c**)		(**b**+**c**)			**a**+**b**+**c**	**c** **b**	**c**+**b**

**Table 4:** Metric relations between the conventional unit cells of lattice types related by normal subgroups, *i.e.* joined by full lines in Fig. 2 ▶

Lattice types L_1_ L_2_	Corresponding space-group types	Basis **a**, **b**, **c** of conventional cell for L_2_ expressed in terms of basis **a**, **b**, **c** of conventional cell for L_1_			Det	Limiting case of L_1_ for which it becomes L_2_	Basis **a**, **b**, **c** of conventional cell for L_1_ expressed in terms of basis **a**, **b**, **c** of conventional cell for L_2_		
		**a**	**b**	**c**			**a**	**b**	**c**
*oP* *tP*	*Pmmm* *P*4/*mmm*	**a**	**b**	**c**	1	*b* = *a*	**a**	**b**	**c**
*oC* *tP*	*Cmmm* *P*4/*mmm*	(**a**+**b**)	(**a**+**b**)	**c**		*b* = *a*	**a** **b**	**a**+**b**	**c**
*oF* *tI*	*Fmmm* *I*4/*mmm*	(**a**+**b**)	(**a**+**b**)	**c**		*b* = *a*	**a** **b**	**a**+**b**	**c**
*oI* *tI*	*Immm* *I*4/*mmm*	**a**	**b**	**c**	1	*b* = *a*	**a**	**b**	**c**
*mP* *oP*	*P*112/*m* *Pmmm*	**a**	**b**	**c**	1	= 90	**a**	**b**	**c**
	*P*12/*m*1 *Pmmm *	**a**	**b**	**c**		= 90	**a**	**b**	**c**
	*P*12/*m*1 *Pmmm*	**b**	**c**	**a**		= 90	**c**	**a**	**b**
*mP* *oS*	*P*112/*m* *Cmmm*	**a**+**b**	**a**+**b**	**c**	2	*b* = *a*	(**a** **b**)	(**a**+**b**)	**c**
*mS* *oS*	*C*12/*m*1 *Cmmm*	**a**	**b**	**c**	1	= 90	**a**	**b**	**c**
*mS* *oS*	*C*12/*m*1 *Cmmm*	**b**	**a**	**c**	1	= 90	**b**	**a**	**c**
*mS* *oF*	*A*112/*m* *Fmmm*	**2a**+**b**	**b**	**c**	2	cos = *b*/(2*a*)	(**a** **b**)	**b**	**c**
	*C*12/*m*1 *Fmmm*	**a**	**b**	2**c**+**a**		cos = *a*/(2*c*)	**a**	**b**	(**c** **a**)
	*C*12/*m*1 *Fmmm*	**b**	**a**	2**c**+**a**		cos = *c*/(2*b*)	**b**	**a**	(**b**+**c**)
*mS* *oI*	*A*112/*m* *Immm*	**b** **a**	**a**	**c**	1	cos = *a*/*b*	**b**	**a** **b**	**c**
	*C*12/*m*1 *Immm*	**c**	**b**	**a** **c**		cos = *c*/*a*	**c** **a**	**b**	**a**
	*C*12/*m*1 *Immm*	**b**	**c**	**a**+**c**		cos = *b*/*c*	**b**+**c**	**a**	**b**
*aP* *mP*	*P* 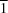 *P*12/*m*1	**a**	**b**	**c**	1	= = 90	**a**	**b**	**c**
	*P* 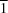 *P*112/*m*	**a**	**b**	**c**		= = 90	**a**	**b**	**c**
*aP* *mS*	*P* 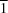 *C*12/*m*1	**a**+**b**	**a**+**b**	**c**	2	*b* = *a*, =	(**a** **b**)	(**a**+**b**)	**c**
	*P* 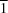 *A*112/*m*	**a**	**b**+**c**	**b**+**c**		*b* = *c*, =	**a**	(**b** **c**)	(**b**+**c**)
